# Ambulatory blood pressure monitoring and blood pressure control in patients with coronary artery disease—A randomized controlled trial

**DOI:** 10.1016/j.ijchy.2020.100074

**Published:** 2020-12-20

**Authors:** Oscar Hägglund, Per Svensson, Cecilia Linde, Jan Östergren

**Affiliations:** a Functional Area of Emergency Medicine, Karolinska University Hospital Solna, Stockholm, Sweden; b Department of Medicine, Solna, Clinical Medicine Unit, Karolinska Institutet, Stockholm, Sweden; c Heart and Vascular Theme Karolinska University Hospital Solna, Stockholm, Sweden; d Department of Medicine, Solna, Cardiology Unit, Karolinska Institutet, Stockholm, Sweden; e Department of Clinical Science and Education, Södersjukhuset, Karolinska Institutet, Sweden; f Department of Cardiology, Södersjukhuset, Sweden

**Keywords:** Blood pressure, Coronary artery disease, Risk factor control, Ambulatory blood pressure, Hypertension

## Abstract

Office blood pressure (OBP) is used for diagnosing and treating hypertension but ambulatory blood pressure measurement (ABPM) associates more accurately with patient outcome. BP control is important in secondary prevention but it is unknown whether the use of APBM improves BP-control in this setting. *Our objective was* to investigate whether physician awareness of ABP after percutaneous coronary intervention (PCI) improved BP-control. *Methods*: A total of 200 patients performed ABPM before and after their PCI follow-up visit. Patients were randomized to open (O) or concealed (C) ABPM results for the physician at the follow-up visit. The change in ABP and antihypertensive medication in relation to baseline ABP was compared between the two groups. *Results*: The average OBP (O and C: 128/76 mmHg) and ABP (O: 123/73 mmHg, C: 127/74 mmHg) was well controlled and did not change between the first and second measurement. A slight increase in systolic ABP during night time was observed in the open arm compared to the concealed arm. Among patients with high ABP (>130/80 mm Hg) at baseline more patients in the C compared to O group remained with a high ABP at the end of study 34/44 (77%) vs 19/34 (56%), p = 0.045. There was a positive correlation between baseline systolic ABP and ABP change in both the O (r = 0.41, p < 0.001) and the C (r = 0.24, p = 0.014) groups but the association was steeper in the open group (p = 0.035). In patients with low ABP an increase and in patients with high ABP a decrease in ABP was observed in the O group where more changes in medication were done. *Conclusions:* ABPM did not lower blood pressure in patients with CAD apart from in those with elevated ABP but led to more relevant changes in antihypertensive treatments. Further studies are needed to answer whether patient outcome is affected.

## 1 Introduction

Hypertension is one of the major risk factors for cardiovascular disease globally [1], but may not be easy to regulate to an optimal blood pressure (BP) level. Traditionally, BP measured in the physician's office (OBP) serves as a basis for diagnosing, monitoring and treatment but ambulatory blood pressure measurement (ABPM) during 24 h gives more accurate information of BP in the patient's daily life [2]. Consequently, ABP has a stronger association with target organ damage [3] and cardiovascular outcomes [4], than OBP. In the 2018 European guidelines ABPM is recommended as an alternative to OBP measurement for hypertension diagnosis [5].

Several studies report a high prevalence of hypertension in patients with cardiovascular disease (CVD) including coronary artery disease (CAD) [6, 7]. However achieving target BP as defined in guidelines is dismal according to registry data. [7], There is limited research regarding ambulatory blood pressure (ABP) in CAD or CVD-patients and such studies are usually descriptive and small in size [[8], [9], [10], [11], [12], [13]], although intervention that lowered night ABP in CVD-patients was associated with improved outcome [14]. The presence of isolated office hypertension, isolated ambulatory hypertension and drug effects on 24 h blood pressure in this large and important group of patients is thus largely unknown. Overall, very few studies have evaluated if an ABP-based strategy improves BP-control [15] and in particular there are no controlled studies on the use of ABPM in patient with CAD. The aim of this randomized study was therefore to investigate whether using ABPM results in routine clinical care improved BP control over 12 weeks in CAD patients after a percutaneous coronary intervention (PCI). The primary outcome measure was the difference in change of night blood pressure between the groups from the first to the second ABPM. A prespecified secondary outcome was the percentage of patients with an initially elevated 24-h BP that achieved a 24-BP within normal limits at the end of study.

## 2 Methods

### 2.1 Study design and patient population

We included 225 patients scheduled for follow up after an acute or elective percutaneous coronary intervention (PCI) at the Department of Cardiology at the Karolinska University Hospital 2009–2015. Exclusion criteria were age <18 or >90 years, cognitive dysfunction, severe somatic disease or current atrial fibrillation. Information on medical history, date and indication for PCI were collected from the medical records, by the study physician (OH).

All patients were subject to ABPM at baseline (3–6 weeks after PCI) and follow up (11–18 weeks after PCI. At the time of the first ABPM the patients also completed a questionnaire about smoking habits and current antihypertensive drug treatment. Weight (kg) and height (cm) was measured. At this visit patients were randomized to one of the two groups (open or concealed ABP). Study staff, patients and the involved physician were aware of their randomization status. At the clinical follow up visit one to two weeks later, which included a clinical OBP-measurement, they were assessed by a cardiologist not involved in the study. At this visit ABPM results were, according to randomization, either used (open group) or not (concealed group) in the decision making for adjustments in antihypertensive medication. Together with the open ABPM-results, the cardiologists were provided with the following reference values: mean 24-h ABP: 130/80 mm Hg; mean daytime ABP: 135/85 mmHg and mean night-time ABP: 120/70 mmHg, according to current guidelines [16,17]. For those with concealed ABPM-results the clinical OBP at the visit was used for decision making regarding changes in antihypertensive treatment. Finally, results regarding BP control were assessed at the second ABPM 8–12 weeks following the first measurement.

### 2.2 Office blood pressure measurement

Immediately before the start of each of the two ABP-monitoring periods, an OBP (referred to as study OBP), was recorded in both arms by a biomedical scientist or a specialized nurse (study staff) using a mercury sphygmomanometer with the subject in the supine position after 5 min of rest. The same study staff was used during the entire length of the study. The mean of two consecutive readings was calculated. If there was a difference in systolic or diastolic BP (SBP, DBP) between the arms of >10 mmHg, the arm with the highest reading was used when defining OBP, otherwise the non-dominant arm was used. The same arm was used at the clinical follow up visit where either the physician or a nurse measured a clinical OBP after having been given instructions for standardized BP measurement as described above. This measurement is referred to as clinical OBP.

### 2.3 Ambulatory BP

Ambulatory BP values were obtained using a noninvasive oscillometric system (Spacelabs 90,217, Spacelabs Healthcare, Hertford, UK). The device was fitted to the patient by one of the study staff. Patients were instructed not to restrict their daily activities during the monitoring periods. Before the start of the monitoring period, the automatic readings were cross-checked against manually measured BP by auscultation. The device was fitted to the nondominant arm, unless a difference of >10 mmHg in SBP between the arms was recorded, in which case the arm with the highest pressure was used. BP and heart rate were recorded automatically every 20 min' for the full 24-h period or in one site (48 patients) every 20 min’ during daytime and every hour at night for a 24-h period. The BP data was auto-edited by the Spacelabs program, which excluded presumably erroneous data. No manual editing of data was carried out in order not to induce bias. Means were calculated for the whole 24-h period, and for day (07.00–21.00) and night (24.00–06.00) periods separately.

### 2.4 Antihypertensive drug treatment

The agents according to guidelines^16 17^ classified as BP lowering were thiazide- and potassium saving diuretics, beta-blockers, calcium antagonists, ACE inhibitors, angiotensin II receptor blockers and others (doxazosin only one used). At the follow up antihypertensive treatment changes were reported by the physician in the study protocol. The patients’ current antihypertensive treatment was also documented prior to the 2nd ABPM by the study staff. All antihypertensive treatment was further recorded as a percentage of maximum recommended daily dose (MRDD), to allow for calculation of treatment change. The latter was calculated as the difference in antihypertensive treatment between prior to the physician follow up and ongoing medication at the 2nd ABPM. Treatment changes as a percentage of MRDD were calculated for each class of antihypertensive agents separately and thereafter changes in all agents were summed together for each patient. Antihypertensive treatment changes were thereafter categorized in the following three groups: dose increased (>0% MRDD), dose unchanged or dose decreased (<0% MRDD).

### 2.5 Statistical analysis

Mean and standard deviation (SD) were used for numerical data whereas median and range was used for the number of days until follow-up visit and the number of BP-lowering agents. Chi-square**-**tests or Fishers exact test were used to compare ratios between groups where the variables were nominal. Independent t-tests were used to compare continuous variables between groups, since the variables investigated were normally distributed. In the subgroup with a high baseline ABP (defined as 24-h BP over 130/80 mm Hg) the number of patients that remained with an uncontrolled 24-h BP was compared between the two groups. In order to study whether the intervention resulted in different BP-responses across the distribution of baseline ABP, analysis of covariance was performed using change in 24-h systolic ABP as the dependent variable and 24-h systolic baseline ABP and study group as independent variables in which the product term between study group and 24- hour systolic ABP was tested. A similar analysis was performed using change in antihypertensive therapy as the dependent variable. Further, changes in intensity of antihypertensive therapy categorized to dose increased, unchanged or decreased was compared between those with an optimal (<115/75 mm Hg), normal and elevated 24-h BP (>130/80 mm Hg) at baseline in the open and concealed group separately [18]. A p-value <0.05 was used to define statistical significance. Statistical analysis was done with TIBCO Software Inc. (2018) Statistica (data analysis software system), version 13. http://tibco.com..%ae.

### 2.6 Ethical considerations

Ethical approval was applied for and approved by the Stockholm Regional Ethical Review Board, reference number 2008/1017–31. All subjects gave informed consent.

The study has ClinicalTrials.gov identifier: NCT04649463

## 3 Results

A total of 225 patients were randomized in a 1:1 fashion to open or concealed ABPM, out of which 25 did not complete the study protocol and were excluded leaving a total of 200 patients for the final analysis. At baseline there was no difference in clinical characteristics between groups except for more patients undergoing an acute PCI for STEMI in the open group and more diabetes type 2 in the concealed group (Table 1). Average OBP and mean ABP were normal in both groups.

**Table 1:** Clinical characteristics and baseline antihypertensive treatment in the concealed (n = 99) and the open (n = 101) groups.

	Concealed	Open	p
**Demography**			
Age (yrs)∗	65 ± 9	65 ± 10	0.989
Gender, Male†	82 (83)	84 (83)	0.949
**PCI-indication**			
STEMI	20 (20)	37 (36)	0.033
NSTEMI/UA	38 (38)	33 (33)	
SA	41 (41)	31 (30)	
**Risk factors and medical history**			
Never smoked	37 (37)	39 (38)	
Previous smoker	53 (54)	55 (55)	
Current Smoker	9 (9)	7 (7)	0.852
Previous AMI	24 (24)	18 (18)	0.265
History of heart failure	6 (6)	7 (7)	0.802
Diabetes Mellitus	31	16 (16)	0.010
BMI (kg/m2)	28 ± 4	27 ± 4	0.162
Hypertension	71 (72)	60 (60)	0.067
**Antihypertensive treatments**			
Diuretics	13 (13)	15 (15)	0.726
Betablockers	92 (93)	94 (93)	0.969
ACE-I	52 (53)	54 (53)	0.894
CA-blockers	22 (22)	24 (24)	0.796
ARB	29 (29)	18 (18)	0.056

Data are presented as mean ± SD or *n* (%). In case of missing values, the total numbers are expressed within the brackets. ABP = ambulatory blood pressure; ACE-I = angiotensin converting enzyme inhibitors; AMI = Acute myocardial infarction; ARB = angiotensin receptor blockers; BMI = body mass index; BP = blood pressure; CA-blockers = calcium channel blockers; Diuretics = potassium saving and thiazide-diuretics; NSTEMI = non-ST-elevated myocardial infarction; SA = stabile angina pectoris; STEMI=ST-elevated myocardial infarction; UA = unstable angina pectoris.

**Fig. 1:**
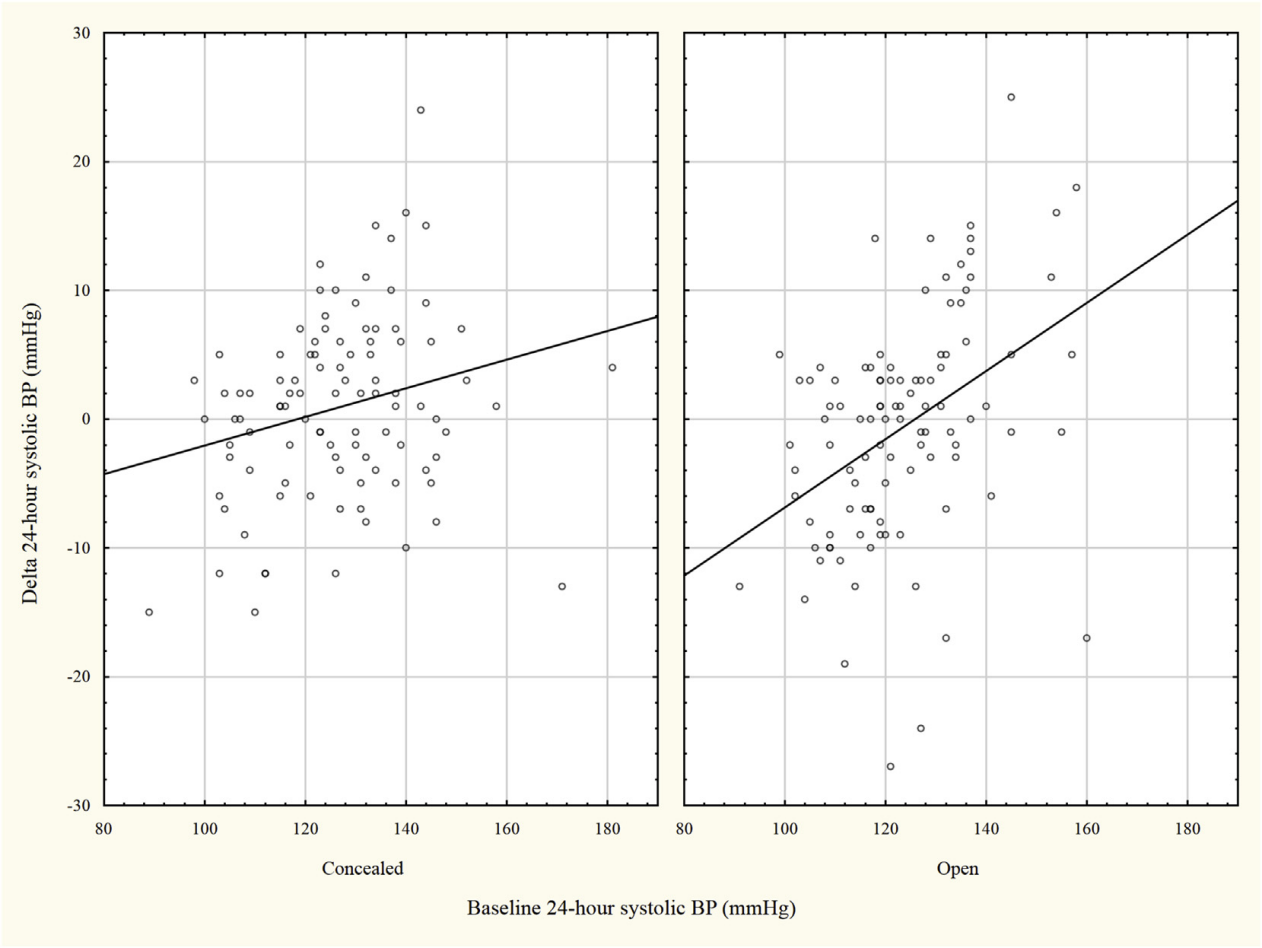
The change of 24-h SBP in relation to baseline 24-h SBP in the two groups. The slope of the regression line was steeper in the open compared to the concealed group (p-value for interaction = 0.035).

A slight increase in systolic ABP during night time was observed in the open arm compared to the concealed arm. Otherwise, the mean differences in ABP between the 1st and 2nd visit indicated no overall change (Table 2) and no differences were seen between the open (O) and concealed (C) arm. In contrast, among patients with a high 24-h ABP at baseline more patients in the concealed compared to the open group remained with a high 24-h ABP at the end of study 34/44 (77%) vs 19/34 (56%), p = 0.045, indicating that ABPM was useful in this group (Table 2). Overall, the change in ABP in relation to baseline ABP differed between the two groups (O: r = 0.41, p < 0.001; C: r = 0.24, p = 0.014) with a steeper regression line in the open group (p-value for interaction = 0.008) (Fig. 1**).** Patients with a lower ABP randomized to the open group had a higher increase in ABP (Fig. 1**)** compared to corresponding patients with concealed ABP. Furthermore, those in the open group with a high baseline ABP decreased in ABP in the second reading compared to the concealed group resulting in different BP responses in the open group across the range of baseline ABP.

**Table 2:** Baseline and follow-up blood pressure data in the concealed (n = 99) and the open (n = 101) groups.

	Concealed	Open	p
**Baseline BP (week 0)**			
24-h BP, mmHg	127 ± 16/74 ± 9	123 ± 14/73 ± 9	0.093/0.481
Daytime BP, mmHg	130 ± 15/76 ± 9 [98]	127 ± 14/76 ± 9	0.133/0.525
Night-time BP, mmHg	118 ± 20/66 ± 12 [97]	114 ± 16/66 ± 9 [100]	0.142/0.958
**Clinical follow-up visit (week 1**–**2)**			
Clinical Office BP, mm Hg	133 ± 16/77 ± 10	132 ± 16/77 ± 10	0.682/0.850
**End of study (week 8**–**12)**			
24-h BP, mmHg	126 ± 16/73 ± 9	124 ± 13/73 ± 8	0.487/0.629
Daytime BP, mmHg	129 ± 15/76 ± 8 [98]	127 ± 13/76 ± 9	0.361/0.862
Night-time BP, mmHg	116 ± 20/66 ± 10 [98]	116 ± 17/67 ± 9 [100]	0.814/0.445
Ambulatory hypertension∗	36 (37)	23 (23)	0.031
24-h hypertension∗∗	38 (38)	26 (26)	0.055
Refractory 24-h hypertension	34 (77) [44]	19 (56) [34]	0.045
**Change in BP between first and second measurement**			
24 h BP, mmHg	1 ± 7/1 ± 5	−1±10/-1±6	0.078/0.064
Daytime BP, mmHg	1 ± 8/0 ± 4 [98]	0 ± 9/0 ± 6	0.296/0.417
Night-time BP, mmHg	1 ± 9/0 ± 9 [97]	−2±10/-1±7 [99]	0.035/0.508

Data are presented as mean ± SD or n (%). In case of missing values or subgroup analysis, the total numbers are expressed within the brackets. ∗ Ambulatory hypertension is defined as average daytime SBP>135 mm Hg or 24-h DBP >85 mm Hg. ∗∗24-h hypertension is defined as average 24-h SBP>130 mm Hg or 24-h DBP >80 mm Hg. Refractory 24-h hypertension is defined as those with 24- hour hypertension on both measurements and the denominator is 24-h hypertension at baseline.

### 3.1 Antihypertensive treatment

The antihypertensive treatment is shown in Table 3. In total there were 31 treatment changes in the concealed group vs. 36 in the open group at the physician follow-up and before the 2nd ABPM. Dose changes in relation to ABP differed between the two groups. In the concealed ABPM group, no association was observed between baseline ABP and dose changes in antihypertensive treatment.

**Table 3:** Antihypertensive daily dose treatment between visits in the two groups. Clinical visit DD = average daily dose of an antihypertensive medicine the patient is treated with at the time of the physician follow-up clinical visit calculated as a percentage of the maximal recommended dose for hypertension, where 1 = maximal daily dose; 2nd ABPM DD = Same as Clinical visit DD but at the time of the second ambulatory blood pressure reading; Change = daily dose of an antihypertensive medicine at the time of the second ambulatory blood pressure reading minus the daily dose of an antihypertensive medicine at the time of the physician follow-up; Total change = the cumulative change in daily dose for all groups of antihypertensive medicines.

	Concealed (n = 99)	Open (n = 101)
**Diuretics**		
Clinical visit DD (%)	4.5	5.5
2nd ABPM DD (%)	5.2	5.9
Change DD (%)	0.8	0.4
**Beta-blockers**		
Clinical visit DD (%)	35.5	43.5
2nd ABPM DD (%)	37.1	40.1
Change DD (%)	1.6	−3.4
**Ace-inhibitors**		
Clinical visit DD (%)	28.1	37.9
2nd ABPM DD (%)	31.8	38.6
Change DD (%)	3.6	0.8
**Calcium-channel blockers**		
Clinical visit DD (%)	16.0	16.8
2nd ABPM DD (%)	15.2	18.2
Change DD (%)	−0.8	1.5
**ARBs**		
Clinical visit DD (%)	17.2	15.0
2nd ABPM DD (%)	16.5	16.2
Change DD (%)	−0.8	1.2
**Total change**	4.5	0.5

ACE-I = angiotensin converting enzyme inhibitors; ARB = angiotensin receptor blockers; Ca-blockers = calcium channel blockers; Diuretics = potassium saving and thiazide-diuretics.

**Fig. 2:**
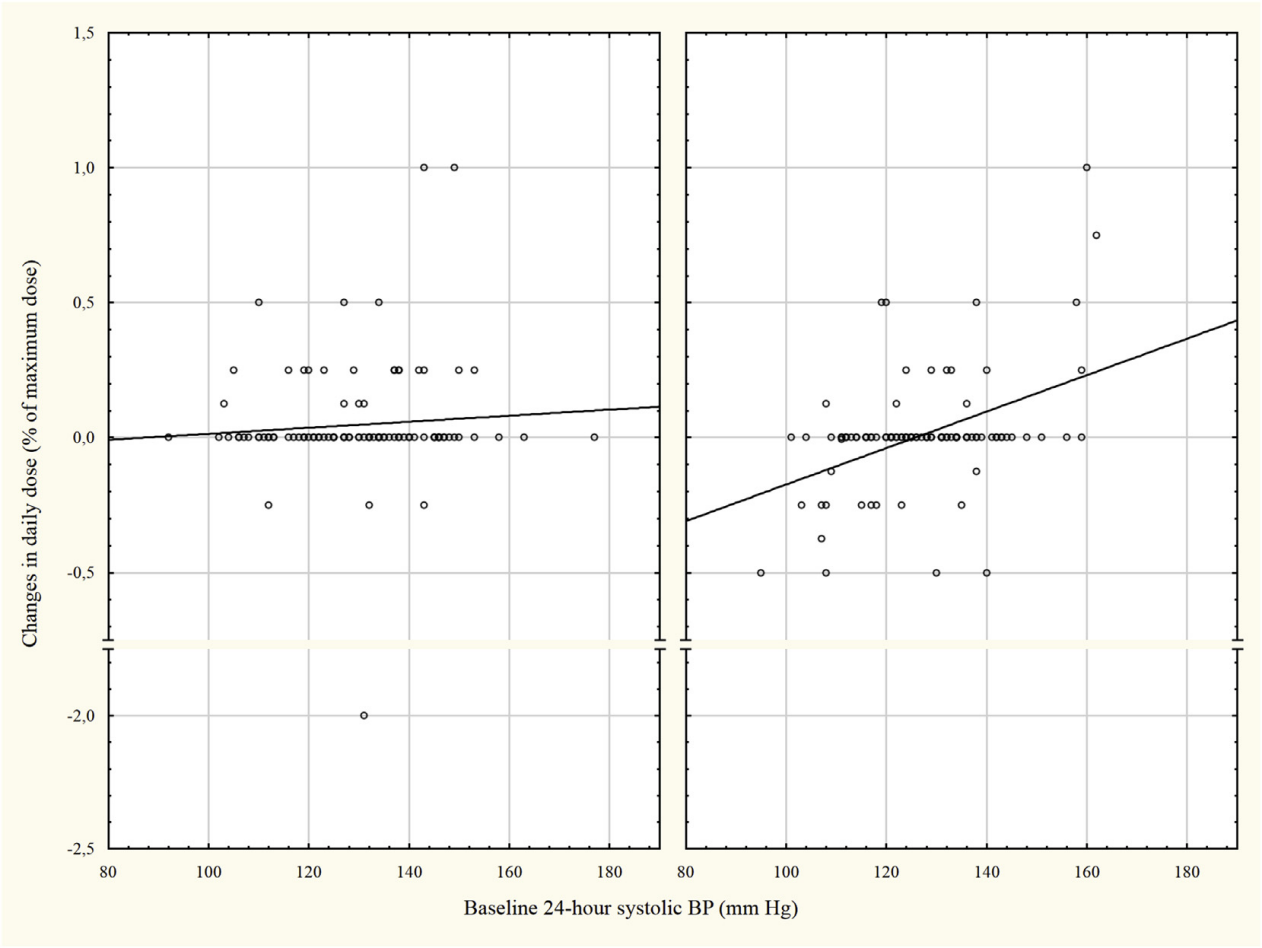
The change of antihypertensive treatment in relation to baseline 24-h SBP in the two groups. No correlation between change and BP was observed in the concealed group but in the open group an association was observed (r = 0.43, p < 0.001).

In contrast, in the open ABPM group a clear association between baseline ABP and dose changes in antihypertensive treatment was observed (r = 0.43, p < 0,001). In those with low baseline ABP there was a decrease in treatment and in patients with a high baseline ABP an increase in treatment (Fig. 2) and Fig. S1. Overall, the change in antihypertensive treatment in relation to baseline ABP differed between the open and concealed groups (p-value for interaction = 0.014)**.**

## 4 Discussion

In this study in patients with coronary artery disease and on average normal blood pressure, ambulatory blood pressure was not further lowered as the result of a randomized intervention using ABP-results in the routinely clinical follow-up after PCI. Conversely, an unexpected minimal increase of average night BP was observed in the group where information on ABP was available. We did however find that ABPM helped to improve BP control since more patients with an initially high ABP had normalized their blood pressure at the end of the study in the group where information on ABP was available. Further, we found an association between baseline ABP and dose changes in antihypertensive drugs in the open group indicating that awareness of ABP had influenced treatment decision making.

In earlier studies it has been found that a high BP in patients with CAD received poor attention [19,20]. Thus, our primary hypothesis was that among patients with uncontrolled ambulatory hypertension the use of ABPM would add valuable information resulting in an enhanced control of ABP and overall lowering of ABP. Contrary to our expectations our study patients generally had a well-controlled ABP suggesting an improvement in adherence to CAD guidelines [7] and it was thus not surprising that the primary endpoint of a lower mean night ABP in patients with an open ABPM reading was not met. Interestingly though and in line with our hypothesis, we found that among patients with a high baseline ABP significantly more patients in the group with open ABPM achieved a normal ABP in the second reading meaning that adjustments of the antihypertensive therapy in the open arm were more relevant than in the concealed arm.

Although numerous studies have shown that ABP is a better predictor for cardiovascular events compared to OBP [4] we are aware of few studies that have studied the effect of using ABP compared to OBP on BP-control and medication. In a randomized controlled trial in 419 untreated hypertensive patients Staessen et al. showed that ABP-guided compared to OBP-guided treatment did not improve BP-control but led to less intense drug treatment [15]. Our findings are in line with this study in that we observed no major difference in BP-control with the ABP-guided therapy but somewhat less intense treatment.

In the open group in our study an association between baseline ABP and dose changes in antihypertensive treatment was found. Patients with very low baseline ABP had their antihypertensive medications reduced and patients with high baseline ABP had an increase in their antihypertensive treatment. In the concealed group where the physicians only had access to OBP no such association was seen. Although our study thus suggests a benefit of using ABPM compared to using only OBP for patients with a high ABP, a caution can be raised for those with a lower ABP among which antihypertensive medication was reduced. It is possible that a less intense BP-control in this group may result in a poorer outcome and more cardiovascular events in the long-term [21]. On the other hand the antihypertensive medication that was most often reduced in the open group with low BP was beta-blockers indicating that the less intense BP-control was driven by a less intense beta-blocker treatment. Recent observational data suggest that treatment with lower doses of beta-blockers are not associated with a poorer outcome after MI [22] and several studies are currently evaluating their routinely use after MI given the lack of clear evidence in post MI patients without heart failure [23].

Further studies are needed to answer whether patient outcome is affected using ABPM in this population. Such intervention studies may include the use of biomarkers in addition to ABP for decisions on treatment since biomarkers add predictive value to ABP [24].

### 4.1 Study limitations

To our knowledge this is the first randomized controlled study on the effect of using ABPM in patients with CAD and this design is a major strength. Another strength is that we used the same study staff during the entire length of the study. The phenomenon of regression to the mean with the probability of the value of the second reading being closer to the average value is probably of limited importance in our study since patients were not selected for the study based on their blood pressure. Further, we believe the risk of this error is less with ABPM compared to OBP.

We were not able to control that the physicians used the ABPM results in their decision on the patients’ treatment, but the differences regarding changes of medication and normalization of ABP in the open group support that they were. Another potential bias is in the recruitment process. We cannot exclude that patients who abstained participation in the study might have a different blood pressure profile compared to the study patients. Further our study was conducted in a single centre hospital with two sites in the capital of Sweden and thus the external validity can be questioned. However when comparing our data to those of Swedish quality of care registries our patients characteristics are similar [25], suggesting that results are valid for patients with CAD in general.

## 5 Conclusions

The use of ABPM did not reduce blood pressure further in patients with CAD and on average normal blood pressure although in patients with high ambulatory BP more patients were identified and controlled. Our study indicated that use of ABPM for high-risk patients with CAD has a role in detecting individuals with higher than expected ABP and through better targeted medication change their blood pressure towards normalization. Further studies are needed to answer whether patient outcome is affected using ABPM in this population.

## Credit author statement

Jan Östergren: Conceptualization, Methodology, Reviewing and Editing.

Oscar Hägglund: Data curation, Writing- Original draft preparation.

Cecilia Linde*:* Supervision, Reviewing and Editing.

Per Svensson: Software, Validation, Reviewing and Editing.

## Funding

Grants were received from the Serafimerlasarett foundation, Sweden, as well as from the Functional Area of Emergency Medicine, Karolinska University Hospital, Solna, Sweden.

## Disclosures

The authors declare that there are no conflicts of interest.
