# Supplementary material for: Ambulatory blood pressure monitoring and blood pressure control in patients with coronary artery disease—A randomized controlled trial
Source: Int J Cardiol Hypertens. 2020 Dec 20;8:100074. doi: 10.1016/j.ijchy.2020.100074 (PMC7803061; doi:10.1016/j.ijchy.2020.100074)

**Appendix. Supplementary data**

**AMBULATORY BLOOD PRESSURE MONITORING OPTIMIZES BLOOD PRESSURE CONTROL IN PATIENTS WITH CORONARY ARTERY DISEASE—A RANDOMIZED CONTROLLED TRIAL**

Oscar HÄGGLUND, MD a,b, Per SVENSSON, MD, PhDe,f, Cecilia LINDE, MD, PhD c,d, Jan ÖSTERGREN, MD, PhD a,b

**Table of contents**

[**eTable 1 –** Quality data on ambulatory blood pressure measurement recordings 1](#__RefHeading___Toc2720945)

[**eTable 2 – Office blood pressure data in the concealed and the open groups** 2](#__RefHeading___Toc2720946)

[**eTable 3 –** Blood pressure in the subgroup with acceptable night ABPM (>=7 successful readings) at both measurements in the concealed (n=96) and the open (n=97) groups 3](#__RefHeading___Toc2720946)

[**eTable 4 –** Blood pressure in the subgroup with acceptable night ABPM (>=7 successful readings) at both measurements in the concealed (n=96) and the open (n=97) groups 4](#__RefHeading___Toc2720946)

[Supplemental Figure S1 - The change of antihypertensive treatment in relation to baseline 24-hour SBP in the two groups. 5](#__RefHeading___Toc2720951)

| eTable1. Quality data on ambulatory blood pressure measurement recordings in the two groups reported as median (5%-95%)   |  | Concealed (n=99) | Open (n=101) | | --- | --- | --- | | **Baseline ABPM (week 0)** |  |  | | Total number of BP-readings | 74 (45-78) | 72 (44-78) | | Number of BP-readings day | 53 (38-57) | 52 (36-58) | | Number of BP-readings night* | 21 (7-23) | 21 (7-21) | | Percentage of successful BP-readings (%) | 95 (74-100) | 95 (70-100) | | Duration (hour:min) | 25:04 (24:00-26:20) | 24:40 (23:40-27:29) | | **End of study ABPM (week 8-12)** |  |  | | Total number of BP-readings | 73 (38-78) | 70 (42-78) | | Number of BP-readings day | 53 (30-57) | 51 (28-57) | | Number of BP-readings night** | 20 (7-23) | 21 (7-21) | | Percentage of successful BP-readings (%) | 95 (51-100) | 94 (58-100) | | Duration (hour:min) | 25:00(23:58-25:43) | 24:40(23:00-25:43) | |  |  |  |   Data are presented as median (5%-95%). * In a total of 48 patients BP was measured once per hour during night-time and a total of 3 patients had less than 7 readings. **2 patients had less than 7 readings |
| --- | --- | --- | --- | --- | --- | --- | --- | --- | --- | --- | --- | --- | --- | --- | --- | --- | --- | --- | --- | --- | --- | --- | --- | --- | --- | --- | --- | --- | --- | --- | --- | --- | --- | --- | --- | --- | --- | --- | --- | --- | --- | --- |

eTable2**.** Office blood pressure data in the concealed (n=99) and the open (n=101) groups.

|  | Concealed | Open | **p** |
| --- | --- | --- | --- |
| **Baseline BP (week 0)** |  |  |  |
| Study OBP, mmHg | 127±15/75±9 | 128±16/75±11 | 0.784/0.536 |
| **End of study (week 8-12)** |  |  |  |
| Study OBP, mmHg | 128±18/76±10 | 128±19/76±10 | 0.873/0.774 |
| **Change between first and second measurement** |  |  |  |
| Study OBP , mmHg | -1±13 /-1±8 | 0±16/-1±9 | 0.625/0.387 |

Data are presented as mean±SD. OBP was measured by a nurse in the supine position before the start of the ABPM

eTable3. Blood pressure in the subgroup with acceptable night ABPM (defined as >=7 successful readings) at both measurements in the concealed (n=96) and the open (n=97) groups.

|  | Concealed | Open | **p** |
| --- | --- | --- | --- |
| **End of study (week 8-12)** |  |  |  |
| 24-hour hypertension | 37 (39) | 24 (25) | 0.039 |
| Refractory 24-hour hypertension* | 33 (77) [43] | 18 (55) [33] | 0.041 |
| **Change in BP between first and second measurement** |  |  |  |
| 24h BP , mmHg | 1±7 /1±5 | -1±9/-0±6 | 0.0742/0.081 |
| Daytime BP, mmHg | 1±7/1±4 | 0±10/0±6 | 0.164/0.342 |
| Night-time BP, mmHg | 1±9 /0±9 | -2±10 /-1±7 | 0.045/0.560 |
|  |  |  |  |

Data are presented as n (%) or mean±SD. In the subgroup analysis, the total numbers are expressed within the brackets. 24-hour hypertension is defined as average 24-h SBP>130 mm Hg or 24-h DBP >80 mm Hg. Refractory 24-hour hypertension is defined as those with 24- hour hypertension on both measurements and the denominator is 24-hour hypertension at baseline.

**eTable 4**

Antihypertensive daily dose treatment between visits among those with optimal baseline 24-h BP (>115/75 mm Hg) in the two groups.

Optimal

|  | Concealed (n=25) | Open (n=28) |  |
| --- | --- | --- | --- |
| **Diuretics** |  |  |  |
| Clinical visit DD (%) | 3.0 | 2.2 |  |
| 2^nd^ ABPM DD (%) | 2.0 | 2.7 |  |
| Change DD (%) | -1.0 | 0.4 |  |
| **Beta-blockers** |  |  |  |
| Clinical visit DD (%) | 34.1 | 34.9 |  |
| 2^nd^ ABPM DD (%) | 35.1 | 26.9 |  |
| Change DD (%) | 1 | -8.1 |  |
| **Ace-inhibitors** |  |  |  |
| Clinical visit DD (%) | 27.0 | 39.3 |  |
| 2^nd^ ABPM DD (%) | 31.5 | 36.3 |  |
| Change DD (%) | 4.5 | -2.7 |  |
| **Calcium-channel blockers** |  |  |  |
| Clinical visit DD (%) | 4.0 | 1.8 |  |
| 2^nd^ ABPM DD (%) | 4.0 | 1.8 |  |
| Change DD (%) | 0 | 0 |  |
| **ARBs** |  |  |  |
| Clinical visit DD (%) | 9.0 | 1.8 |  |
| 2^nd^ ABPM DD (%) | 9.0 | 2.7 |  |
| Change DD (%) | 0 | 0.9 |  |
| **Total change** | 6.5 | -9.4 |  |

ACE-I=angiotensin converting enzyme inhibitors; ARB=angiotensin receptor blockers; Ca-blockers=calcium channel blockers; Diuretics=potassium saving and thiazide-diuretics. Clinical visit DD =average daily dose of an antihypertensive medicine the patient is treated with at the time of the physician clinical visit. Calculated as a percentage of the maximal recommended dose for hypertension; 2^nd^ ABPM DD= Same as clinical visit DD but at the time of the second ambulatory blood pressure reading; Change=daily dose of an antihypertensive medicine at the time of the second ambulatory blood pressure reading minus the daily of an antihypertensive medicine at the time of the physician follow-up; Total change=the cumulative change in daily dose for all groups of antihypertensive medicines.

##

Supplemental Fig. S1 - The change of antihypertensive treatment in relation to baseline 24-hour BP (optimal; <115/75 mm Hg; normal; >130/80 mm Hg (ambulatory hypertension)) in the two groups. No association between treatment change and ABP was observed in the concealed group (p=0.978) but in the open group an association was observed (p=0.005).


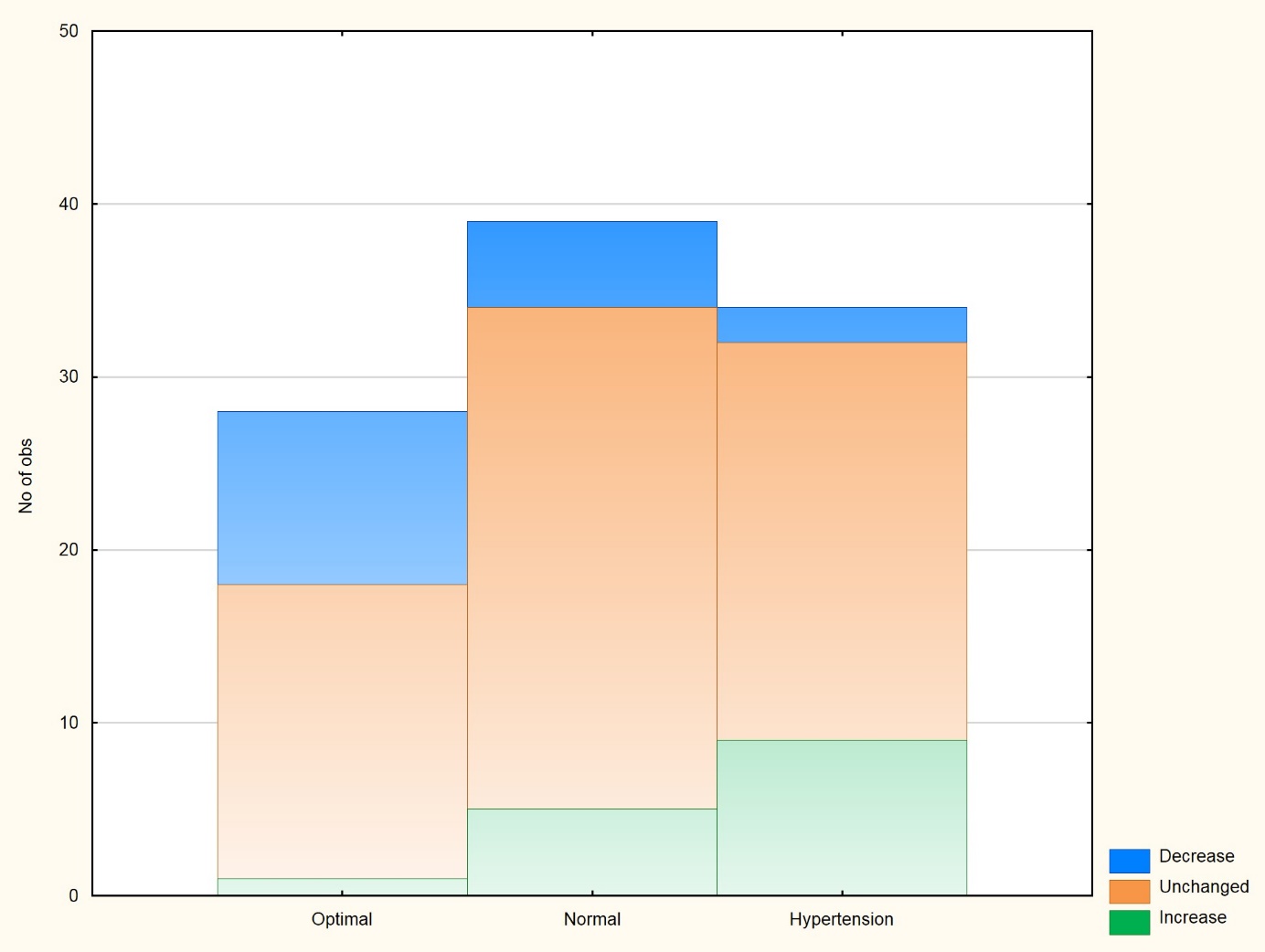


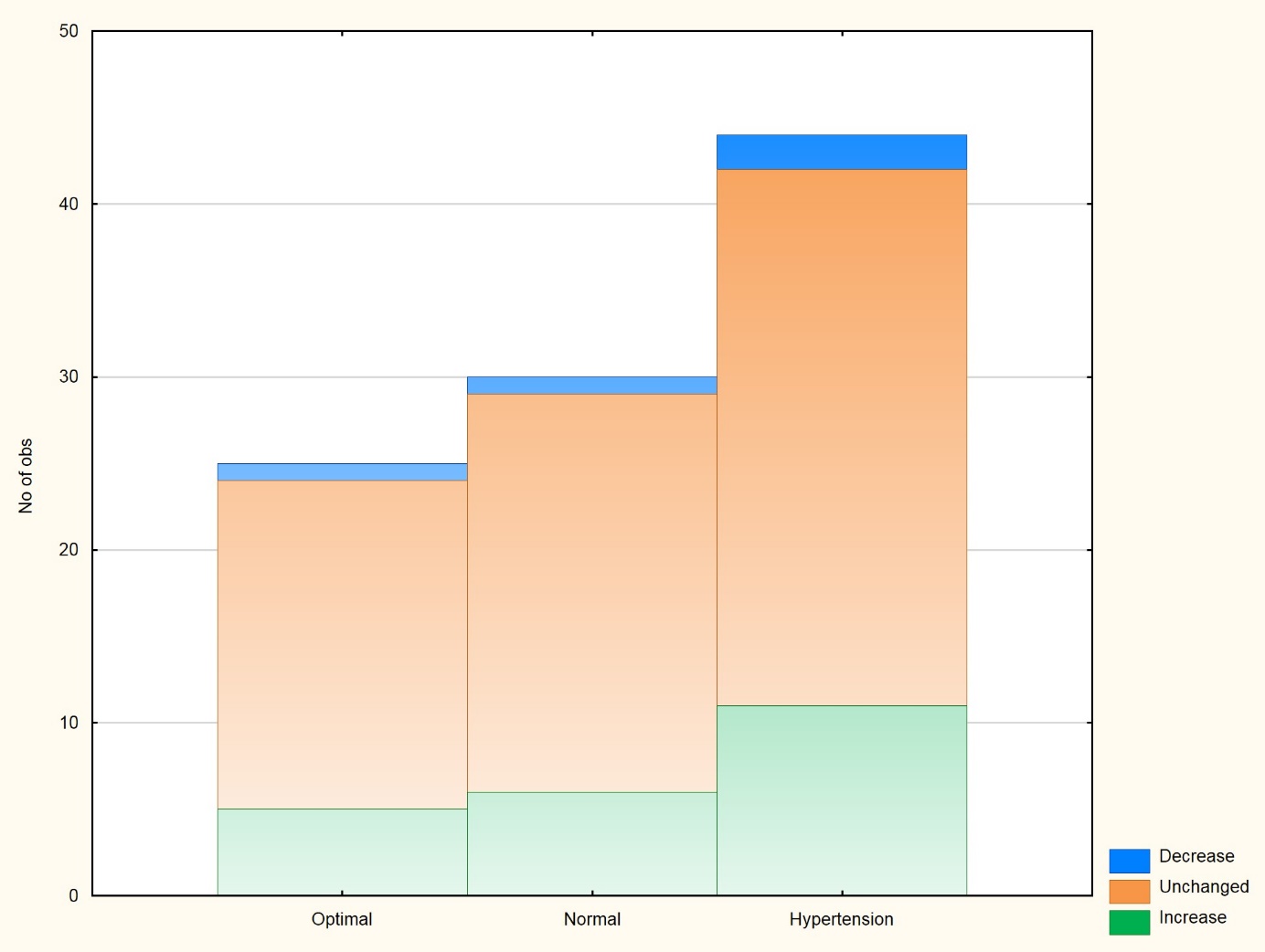

Supplement: Multimedia component 1 [file mmc1.doc]
